# Carvacrol, Thymol, and Garlic Essential Oil Promote Skin Innate Immunity in Gilthead Seabream (*Sparus aurata*) Through the Multifactorial Modulation of the Secretory Pathway and Enhancement of Mucus Protective Capacity

**DOI:** 10.3389/fimmu.2021.633621

**Published:** 2021-03-12

**Authors:** Joana P. Firmino, Laura Fernández-Alacid, Eva Vallejos-Vidal, Ricardo Salomón, Ignasi Sanahuja, Lluis Tort, Antoni Ibarz, Felipe E. Reyes-López, Enric Gisbert

**Affiliations:** ^1^IRTA, Centre de Sant Carles de la Ràpita (IRTA-SCR), Aquaculture Program, Sant Carles de la Ràpita, Spain; ^2^TECNOVIT–FARMFAES, S.L. Pol. Ind. Les Sorts, Alforja, Spain; ^3^Ph.D. Program in Aquaculture, Universitat Autònoma de Barcelona, Bellaterra, Spain; ^4^Department of Cell Biology, Physiology and Immunology, Faculty of Biology, University of Barcelona, Barcelona, Spain; ^5^Departamento de Biología, Facultad de Química y Biología, Centro de Biotecnología Acuícola, Universidad de Santiago de Chile, Santiago, Chile; ^6^Department of Cell Biology, Physiology and Immunology, Universitat Autònoma de Barcelona, Bellaterra, Spain; ^7^Facultad de Medicina Veterinaria y Agronomía, Universidad de Las Américas, Santiago, Chile; ^8^Consorcio Tecnológico de Sanidad Acuícola, Ictio Biotechnologies S.A., Santiago, Chile

**Keywords:** SALT, innate immunity, stress, aquaculture, *Vibrio* infection, teleost fish skin mucus, phytogenic additive, interactome

## Abstract

One of the main targets for the use of phytogenics in aquafeeds is the mucosal tissues as they constitute a physical and biochemical shield against environmental and pathogenic threats, comprising elements from both the innate and acquired immunity. In the present study, the modulation of the skin transcriptional immune response, the bacterial growth capacity in skin mucus, and the overall health condition of gilthead seabream (*Sparus aurata*) juveniles fed a dietary supplementation of garlic essential oil, carvacrol, and thymol were assessed. The enrichment analysis of the skin transcriptional profile of fish fed the phytogenic-supplemented diet revealed the regulation of genes associated to cellular components involved in the secretory pathway, suggesting the stimulation, and recruitment of phagocytic cells. Genes recognized by their involvement in non-specific immune response were also identified in the analysis. The promotion of the secretion of non-specific immune molecules into the skin mucus was proposed to be involved in the *in vitro* decreased growth capacity of pathogenic bacteria in the mucus of fish fed the phytogenic-supplemented diet. Although the mucus antioxidant capacity was not affected by the phytogenics supplementation, the regulation of genes coding for oxidative stress enzymes suggested the reduction of the skin oxidative stress. Additionally, the decreased levels of cortisol in mucus indicated a reduction in the fish allostatic load due to the properties of the tested additive. Altogether, the dietary garlic, carvacrol, and thymol appear to promote the gilthead seabream skin innate immunity and the mucus protective capacity, decreasing its susceptibility to be colonized by pathogenic bacteria.

## Introduction

Fish infectious diseases are one of the main constraints of the aquaculture sector, representing a serious economic, social, and environmental challenge for the industry (1). Since fish farmers depend on high survival rates and healthy animals, strategies to improve their performance, immune status, and welfare are highly demanded for supporting health management practices that positively impact in the final revenue of the fish farm. On this basis, the development of functional feed additives designed to physiologically support fish to cope with pathogenic and other external challenges intrinsic to aquaculture rearing conditions, represents a promising tool to be implemented in a sustainable and environmentally-responsible aquaculture industry (2).

Functional feeds containing essential oils, one of most commonly used group of phytogenics in aquafeeds, have received increased attention during these last years due to their antimicrobial, immunostimulant, antioxidant, anti-stress, and growth-promoting properties (3–5). Besides essential oils being repeatedly demonstrated to stimulate both humoral and cellular components of the fish innate immunity (6), numerous have been also shown to display a noteworthy antimicrobial activity against a wide range of fish pathogens (7, 8), putting them on the spotlight for the development of sustainable prophylactics. Particularly, phytogenics derived from garlic (*Allium sativum* L., Alliaceae, Liliaceae), oregano (*Oreganum vulgare*, Labiateae), and thyme (*Thymus vulgare*, Labiateae) are among the most studied and administrated, due to their recognized health-promoting properties for aquatic species (6).

One of the main targets for this type of nutritional strategies is the mucosal tissues, due to their importance in the protection against the immediate contact with the environment and potential pathogens. Besides acting as a physical barrier, the mucosal layer also offers a biochemical shield, in which elements from both the innate and acquired immunity are present (9). In particular, the fish skin mucus represents the largest mucosal barrier with its whole epidermis directly exposed to the environment. It is responsible for the first line of defense against external threats, determining pathogen adhesion to the epithelial surfaces (10, 11). Furthermore, skin mucus participates in important physiological processes like osmoregulation, swimming, sensory reception (12), and ecological intra and interspecific interactions (11). Additionally, the fish skin is also characterized by its active mucosal immunity, containing a skin-associated lymphoid tissue (SALT), which is able to respond in case of infection (12, 13). In fact, the immune response described in fish skin against antigen stimulation is similar to other mucosa (14). That immune response involves the secretion of innate immune molecules and the action of specialized cells [(15) and references therein].

Therefore, the improvement of both the epidermal mucus composition and the SALT response to environmental stressors including infective agents by means of dietary tools such as functional feed additives, represents a promising approach for preventing bacterial-induced pathologies in farmed fish. In this context, the aim of the present study was to evaluate the inclusion of a functional feed additive composed by a blend of garlic essential oil, carvacrol, and thymol (the main bioactive compounds of Labiateae plants essential oils) in a standard on-growing diet for gilthead seabream (*Sparus aurata*), assessing its effects on the skin transcriptional response, pathogenic bacterial growth capacity in skin mucus, and fish overall health condition. This species was chosen since it is the most important marine farmed fish species in the Mediterranean with an annual production of 85,385.1 t in 2018 and an economic value of 502,398,000 US$ (16); thus, improving health management strategies based on sustainable dietary approaches for this farmed species is of relevance for this growing industry.

## Materials and Methods

### Diets and Fish Rearing

Juveniles of gilthead seabream were purchased from a Mediterranean fish farm (Piscicultura Marina Mediterránea S.L., Andromeda Group, Valencia, Spain) and on-grown at IRTA–Sant Carles de la Ràpita facilities for research purposes (17). Before the onset of the trial, fish were individually measured in body weight (BW) and standard length (SL) to the nearest 0.1 g and 1 mm, respectively (BW = 40.3 ± 0.1 g; SL = 12.0 ± 0.2 mm). Then, 150 juveniles were randomly distributed among six 450 L tanks (25 fish per tank; initial density = 2 kg m^−3^; three replicate tanks per experimental group) connected to an IRTAmar® system working under an open-flow regime.

Fish were fed two experimental diets, one devoid of the functional feed additive (control diet) and a second one supplemented with 0.5% of a microencapsulated functional additive containing synthetic garlic essential oil, carvacrol, and thymol (AROTEC-G®, TECNOVIT-FARMFAES, S.L., Spain). Both diets were tested in triplicate tanks and administered for a period of 65 days. Fish were hand-fed two times per day at the daily rate of 3.0% of the stocked biomass, which approached apparent satiation. The control diet was formulated with high levels of marine-derived protein sources (30% fishmeal, 2.5% soluble protein concentrate—CPSP 90® and 2.5 krill meal), containing 46% crude protein, 18% crude fat, and 21.5 MJ/kg gross energy (Table 1). Both tested experimental diets were formulated to fulfill the nutritional requirements of juvenile gilthead seabream for summer conditions (18). Diets were manufactured by Sparos Lda. (Olhão, Portugal). In particular, main ingredients were ground (below 250 μm) in a micropulverizer hammer mill (SH1; Hosokawa Micron, B.V., Doetinchem, The Netherlands). Powder ingredients and oils were then mixed according to the target formulation in a paddle mixer (RM90; Mainca, S.L., Granollers, Spain). All diets were manufactured by temperature-controlled extrusion (pellet sizes: 2.0 mm) by means of a low-shear extruder (P55; Italplast, S.r.l., Parma, Italy). Upon extrusion, all feed batches were dried in a convection oven (OP 750-UF; LTE Scientifics, Oldham, UK) for 4 h at 45°C.

**Table 1:** Formulation of the control diet used during the nutritional assay.

**Ingredients**	**Control diet (%)**
Fishmeal 70 LT FF Skagen	20.0
Fishmeal CORPESCA Super Prime	10.0
CPSP 90	2.5
Squid meal	2.5
Soy protein concentrate (Soycomil)	5.0
Wheat Gluten	5.0
Corn gluten	8.0
Korfeed 60	4.5
Soybean meal 48	8.0
Rapeseed meal	4.0
Sunflower meal	3.0
Wheat meal	7.0
Whole peas	2.5
Fish oil–COPPENS	9.0
Soybean oil	1.5
Rapeseed oil	2.5
Vitamin and mineral Premix PV01	2.0
Soy lecithin–Powder	2.0
Antioxidant powder (Paramega)	0.4
Dicalcium phosphate	0.6
**Proximate composition, % in dry basis**	
Crude protein	46.2
Crude fat	18.4
Gross energy (MJ/kg)	21.5

The nutritional assay was performed under natural photoperiod (August–September), with daily monitoring of the water temperature (25.1 ± 1.5°C, range: 22.6–28°C), oxygen (6.8 ± 1.7 mg/L; >80% saturation) (OXI330, Crison Instruments, Barcelona, Spain) and pH (7.5 ± 0.01) (pHmeter 507, Crison Instruments). Salinity (35‰) (MASTER-20 T; ATAGO Co. Ltd, Tokyo, Japan), ammonia (0.13 ± 0.1 mg ${\text{NH}}_{4}^{+}$/L), and nitrite (0.18 ± 0.1 mg ${\text{NO}}_{2}^{-}$/L) levels (HACH DR9000 Colorimeter, Hach®, Spain) were weekly measured.

### Sampling

At the end of the trial, all fish were anesthetized (buffered 150 mg/L MS-222, Sigma-Aldrich, Madrid, Spain) and measured for individual body weight and standard length (BW_control diet_ = 157.8 ± 14.2 g and SL_control diet_ = 17.3 ± 0.6 cm; BW_supplemented diet_ = 150.8 ± 14.9 g and SL_supplemented diet_ = 17.1 ± 0.6 cm) as published in (17). Then, eight fish from each tank (*n* = 24 per dietary treatment) were randomly selected and skin mucus sample collected following the method described in (19). In brief, skin mucus was collected from the over-lateral line of anesthetized fish in a front to caudal direction using sterile glass slides, and mucus was carefully pushed and collected in a sterile tube (2 mL), avoiding the contamination with blood and/or urine-genital and intestinal excretions. The above-mentioned procedure lasted <2 min in order to avoid the degradation of mucus metabolites. Mucus samples were homogenized using a sterile Teflon pestle to desegregate mucus mesh before centrifugation at 14,000 × g during 15 min at 4°C. The resultant mucus supernatants were collected, avoiding the surface lipid layer, aliquoted, and stored at −80°C for further analysis. For transcriptional analysis purposes, other four fish were randomly selected from each tank (*n* = 12 fish per dietary treatment) and euthanized with an anesthetic overdose. A ca. 1 cm^2^ section of the skin from the mid region of the body over the lateral line of the right side from each fish was dissected, and the muscle tissue attached to it removed. Samples were immersed in RNAlater™ (Invitrogen, Thermo Fisher Scientific, Lithuania), incubated overnight (4°C) and stored at −80°C for further RNA extraction.

### Skin Transcriptomic Analysis

#### RNA Isolation and Quality Control

Total RNA from the skin of twelve randomly selected fish per dietary treatment was extracted using the RNeasy® Mini Kit (Qiagen, Germany). Total RNA was eluted in a final volume of 35 μL nuclease-free water and treated with DNAse (DNA-free^TM^ DNA Removal Kit; Invitrogen, Lithuania). Total RNA concentration and purity were measured using Nanodrop-2000® spectrophotometer (Thermo Scientific, USA) and stored at −80°C until analysis. Prior to hybridization with microarrays, RNA samples were diluted to 133.33 ng/μL concentration, checked for RNA integrity (Agilent 2100 Bioanalyzer; Agilent Technologies, Spain) and selected by the criteria of a RIN value >8.5. Three different pools of samples per dietary treatment were established (*n* = 4 fish each pool).

#### Microarray Hybridization and Analysis

Skin transcriptional analysis from both experimental groups was carried out using the Aquagenomics *Sparus aurata* oligonucleotide microarray v2.0 (4 × 44 K) (SAQ) platform. Detailed information and transcriptomic raw data are available at the Gene Expression Omnibus (GEO) public repository at the US National Center for Biotechnology Information (NCBI), accession numbers GPL13442, and GSE162504, respectively. The sampling labeling, hybridization, washes, and scanning was performed as described in (19). Briefly, a one-color RNA labeling was used (Agilent One-Color RNA Spike-In kit; Agilent Technologies, USA). RNA from each sample pool (200 ng) was reverse-transcribed with spike-in. Then, total RNA was used as template for Cyanine-3 (Cy3) labeled cRNA synthesis and amplified with the Quick Amp Labeling kit (Agilent Technologies). cRNA samples were purified using the RNeasy® micro kit (Qiagen). Dye incorporation and cRNA yield were checked (NanoDrop ND-2000® spectrophotometer). Then, Cy3-labeled cRNA (1.5 mg) with specific activity >6.0 pmol Cy3/mg cRNA was fragmented at 60°C for 30 min, and hybridized with the array in presence of hybridization buffer (Gene expression hybridization kit, Agilent Technologies) at 65°C for 17 h. For washes, microarrays were incubated with Gene expression wash buffers, and stabilization and drying solution according to manufacturer instructions (Agilent Technologies). Microarray slides were then scanned (Agilent G2505B Microarray Scanner System), and spot intensities and other quality control features extracted (Agilent Feature Extraction software version 10.4.0.0).

The Search Tool for the Retrieval of Interacting Genes (STRING) public repository version 11.0 (https://string-db.org) was used to generate the skin transcripteractome for the fish fed the phytogenic-supplemented diet. A Protein-Protein interaction (PPI) Networks Functional Enrichment Analysis for all the differentially expressed genes (DEGs) was conducted with a high-confidence interaction score (0.9) using *Homo sapiens* as model organism (17, 20). Gene Ontology (GO) and Kyoto Encyclopedia of Genes and Genomes (KEGG) enrichment analysis of all the DEGs obtained were also assessed through STRING (*P* < 0.05). In order to confirm match of gene acronyms between both *Homo sapiens* and gilthead seabream species, human orthology identification based on gene/protein name was accessed through the Genecards (www.genecards.org) (21) and Uniprot (www.uniprot.org) databases. Additionally, protein-protein BLAST (BLASTp) were run (*E* < 10^−7^; query cover > 95%).

### Skin Mucus Parameters

#### Bacterial Growth Assessment in Skin Mucus

Two bacterial fish pathogens were used for the growth curve assay, *Vibrio anguillarum* (CECT number: 522T), and *Pseudomonas anguilliseptica* (CECT number: 899T) from the Spanish Type Culture Collection (CECT, University of Valencia, Valencia, Spain), and the non-pathogenic bacterium for fish, *Escherichia coli* (DSMZ number: 423) from the German Collection of Microorganisms and Cell Cultures (Leibniz Institute DSMZ, Germany). The two pathogenic bacteria were cultured at 30°C for 24 h in marine broth (MB-2216, Becton and Dickinson, USA) and the *E. coli* was cultured at 37°C for 24 h in trypticasein soy broth (TSB, Laboratorios Conda, Spain). For the culture in skin mucus, bacterial suspension with optical density (OD) of 0.2 were centrifuged and the pellet resuspended in sterile PBS, diluted in new growth medium and adjusted to 106 colony-forming units (CFU) mL^−1^. Then, to study the bacterial growth capacity in the skin mucus, aliquots of 100 μL of the previously cultured bacteria were incubated in 100 μL of skin mucus (3 pools of 6 individual fish per dietary treatment). In parallel, 100 μL of the same cultured bacteria were also incubated in 100 μL of its respective culture media, as a positive control. Triplicates of 100 μL of each fish mucus samples added to 100 μL of culture media were used as negative control and values subtracted from the bacteria–mucus aliquot results. The bacterial growth was measured by absorbance at λ = 400 nm every 30 min for 14 h at 25°C in flat-bottomed 96-well plates using an Infinity Pro200^TM^ spectrophotometer. Similar temperature values for bacterial growth cultures (25°C) and fish rearing (25.1°C) were chosen in order to standardize mucus analyses with regard to fish rearing conditions used in the nutritional trial.

#### Skin Mucus Metabolites and Cortisol Analyses

Glucose concentration on fish skin mucus was determined by an enzymatic colorimetric test (LO-POD glucose, SPINREACT®, St. Esteve de Bas, Spain) as described in (19). The OD of the reaction was determined at λ = 505 nm with a microplate reader and glucose values expressed as μg glucose per mL of skin mucus. Lactate concentration was determined by an enzymatic colorimetric test (LO-POD lactate, SPINREACT®) following the manufacturer's instructions but with slight modifications for fish skin mucus (19). The OD was determined at λ = 505 nm and lactate values expressed as μg lactate per mL of skin mucus. Protein concentration of previously homogenized mucus samples was determined using the Bradford assay (22) using bovine serum albumin (Sigma Aldrich, Madrid, Spain) as standard. In particular, mucus samples or standard solutions (from 0 to 1.41 mg mL^−1^) were mixed with 250 μL of Bradford reagent and incubated for 5 min at room temperature. The OD was determined at λ = 596 nm in a microplate reader. Protein values were expressed as mg protein per mL of skin mucus. Cortisol levels were measured using an ELISA kit (IBL International, Tecan Group, Switzerland) following the manufacturer's instructions for saliva determinations. Values of OD were determined at λ = 450 nm with a microplate reader. Cortisol values were expressed as ng cortisol per mL of skin mucus. All standards and samples were analyzed in triplicate (methodological replicates) and spectrophotometric measurements were conducted with an Infinity Pro200^TM^ spectrophotometer (Tecan, Männedorf, Switzerland).

Mucus ratios referred to protein (glucose/protein, lactate/protein and cortisol/protein) were calculated in order to avoid the putative dilution or concentration derived from mucus sampling. As an indicator of the metabolic aerobic response, the glucose/lactate ratio was also calculated (19).

Ferric Antioxidant Power (FRAP) was measured by means of an enzymatic colorimetric test (Ferric antioxidant status detection kit, Invitrogen, Thermo Fisher Scientific, Spain), following the manufacturer's instructions for plasma, with minor modifications. Briefly, 20 μL of mucus sample or standard solutions (from 0 to 1,000 μM μL^−1^ of FeCl_2_) were mixed with 75 μL of FRAP color solution and incubated at room temperature for 30 min, in triplicate. The OD was measured at λ = 560 nm. Antioxidant values were expressed as nmol FRAP per mL of mucus, and nmol FRAP per mg of mucus protein. All measurements were performed with a microplate spectrophotometer reader (Infinity Pro200^TM^ spectrophotometer).

### Statistical Analysis

Differences between growth performance parameters were analyzed through an unpaired *t*-test (*P* < 0.05) with GraphPad PRISM 7.00 assuming data homoscedasticity. Differences between skin mucus metabolites and cortisol, and differences in bacterial growth inhibition between the two dietary treatments were assessed with SPSS Statistics for Windows, Version 22.0 (IBM Corp, Armonk, NY, USA) through an unpaired *t*-test (*P* < 0.05). Microarrays extracted raw data were imported and analyzed with GeneSpring version 14.5 GX software (Agilent Technologies). The 75% percentile normalization was used to standardize arrays for comparisons and data were filtered by expression. An unpaired *t*-test was conducted without correction to identify those DEGs between both dietary treatments. A *P* < 0.05 was considered statistically significant. The representation for the principal component analysis (PCA) and the hierarchical heatmap were generated using GeneSpring version 14.5 GX software.

## Results

### Skin Transcriptomic Profile

**Figure 1:**
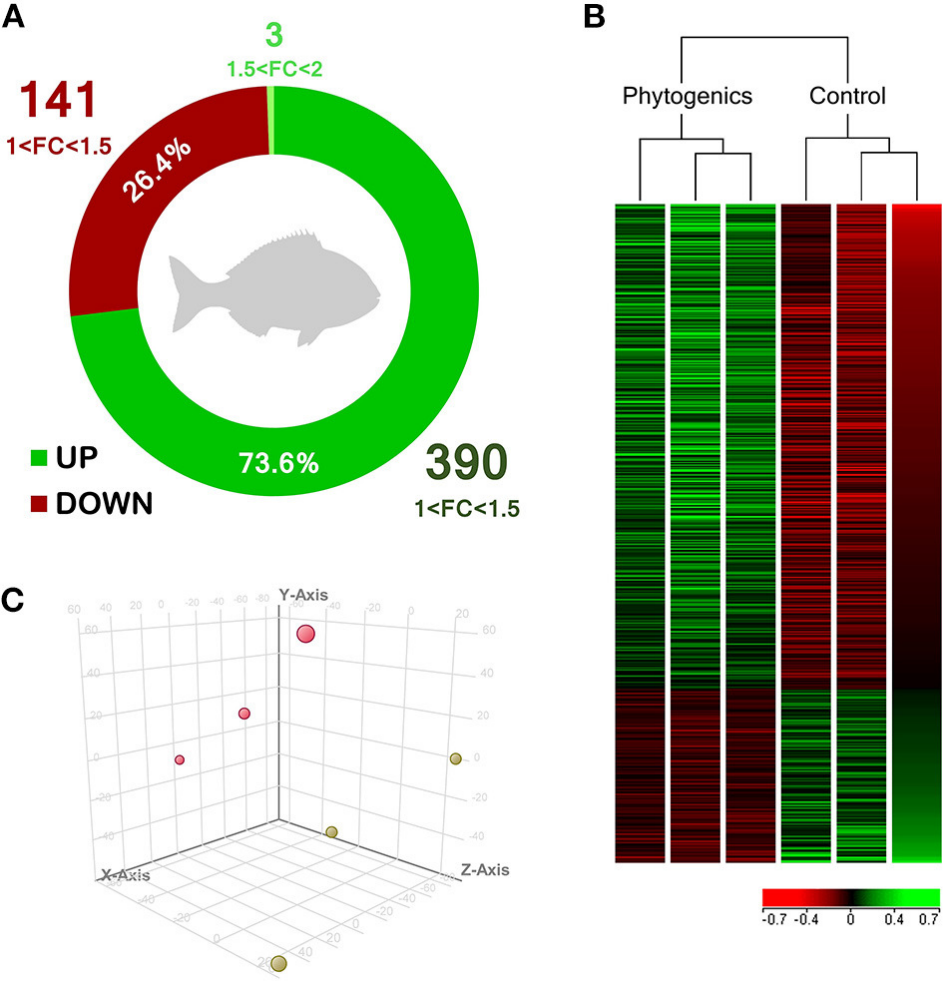
Skin transcriptomic profile of gilthead seabream (*Sparus aurata*) fed a diet supplemented with a blend of garlic essential oil, carvacrol and thymol. **(A)** Differential expression analysis of the gilthead seabream skin transcriptomic response fed a diet supplemented with a blend of garlic, carvacrol and thymol. **(B)** Hierarchical clustering for the control and phytogenic-supplemented diets, based in similitude patterns of the differentially expressed genes (DEGs) detected from three sample pools per dietary group. Data of the six microarrays are depicted, one for each represented pool. Both increased and decreased gene expression pattern is shown in green and red, respectively. All transcripts represented are statistically significant (*P* < 0.05). **(C)** Principal component analysis (PCA) of the DEGs of gilthead seabream skin response to the control diet (yellow) and phytogenic-supplemented diet (pink). Please refers to Supplementary Table 1 for details.

Under present experimental conditions, in order to determine the modulatory effect of the dietary supplementation of a blend of garlic essential oil, carvacrol, and thymol upon the skin transcriptome, a microarray-based transcriptomic analysis was conducted in gilthead seabream. In total, 534 differentially-expressed genes (DEGs) were found in the skin from both experimental groups (*P* < 0.05; Supplementary Table 1). Among these, 393 genes were up-regulated with 390 belonging to the 1.0–1.5-fold change (FC) interval. The other 3 DEGs were grouped in the 1.5 ≤ FC ≤ 2.0 interval. On the other hand, 141 genes were down-regulated (*P* < 0.05) and grouped in the range of−1.5 ≥ FC ≥ −1.0. Although genes were observed to be mostly up-regulated in the group fed with the blend of phytogenics (73.6% of DEGs), gene modulation was moderated in terms of fold-change intensity (Figure 1A). Common segregation among the pool samples within the same dietary treatment was observed in the hierarchical clustering for the skin transcriptomic response based in similitude patterns of the DEGs response (*P* < 0.05) (Figure 1B). The observed differential profile among dietary treatments is supported by the PCA analysis for the analyzed samples (Figure 1C).

**Figure 2:**
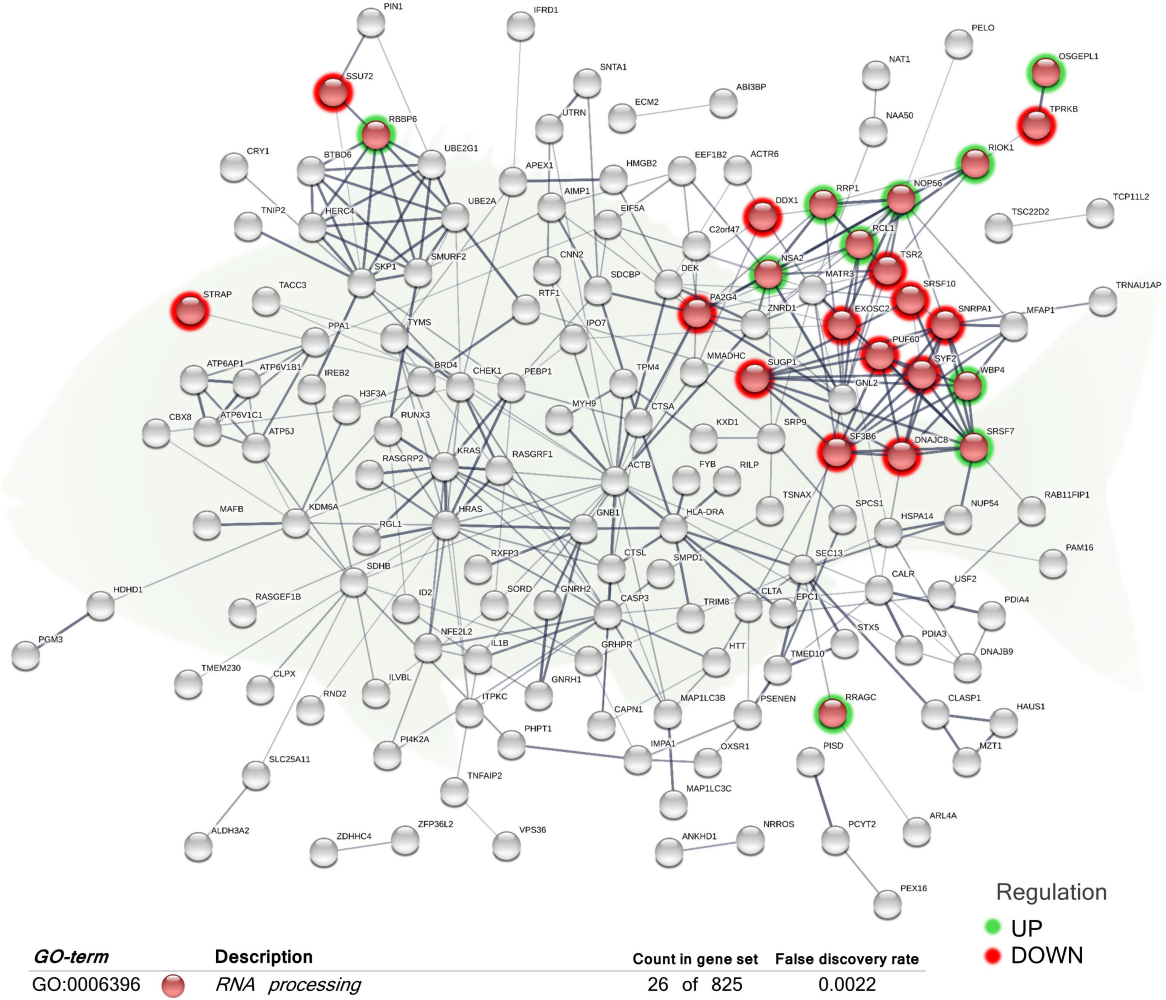
Functional biological network of the differentially expressed genes (DEGs) in the skin of juvenile gilthead seabream (*Sparus aurata*) fed a diet supplemented with a blend of garlic essential oil, carvacrol, and thymol. Green-shaded nodes represent up-regulated genes and red-shaded nodes represent down-regulated genes. Graphic keys and network stats: number of nodes = 203; number of edges = 341; average node degree = 3.36; average local clustering coefficient = 0.4; expected number of edges = 286; PPI enrichment *p*-value = 0.000807. Gene Ontology (GO) definition, count of DEGs within the biological process and respective false discovery rate are described in the graphical figure legend (bottom). For details, please refers to Supplementary Table 2.

**Figure 3:**
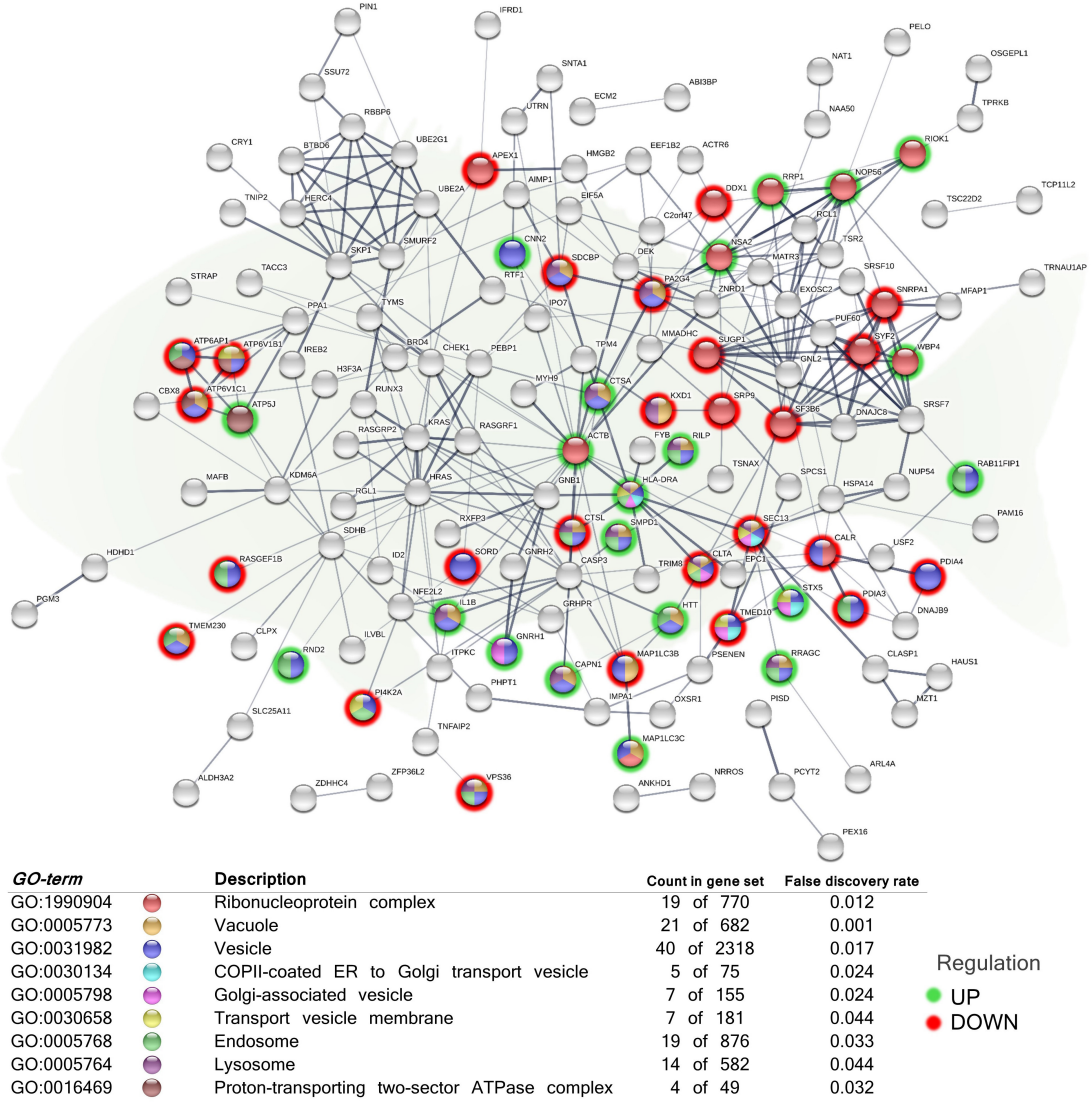
Functional network representing cellular components related to secretory pathways of the differentially expressed genes (DEGs) in the skin of juvenile gilthead seabream (*Sparus aurata*) fed a diet supplemented with a blend of garlic essential oil, carvacrol and thymol. Node colors indicate the cellular component for each DEG represented. Green-shaded nodes represent up-regulated genes and red-shaded nodes represent down-regulated genes. Graphic keys and network stats: number of nodes = 203; number of edges = 341; average node degree = 3.36; average local clustering coefficient = 0.4; expected number of edges = 286; PPI enrichment *p*-value = 0.000807. Gene Ontology (GO) definition, count of DEGs within the biological process and respective false discovery rate are described in the graphical figure legend (bottom). For details, please refers to Supplementary Table 2.

When considering the complete list of annotated DEGs, a functional network (transcripteractome) containing 203 nodes was generated (Figures 2, 3), which resulted in 341 interactions (edges). The remaining 331 DEGs, annotated as unknown genes, were excluded from the analysis. The enrichment analysis identified in the transcripteractome two main representative processes that were considered to encompass the several Gene Ontology (GO) annotations obtained (Supplementary Table 2), denoted as (a) Transcription Regulation, and (b) Secretory Pathway.

The “RNA processing” biological process (GO:0006396; 12 up-regulated genes; 14 down-regulated genes) was the exclusive differentially regulated GO term for the skin of fish fed the diet supplemented with the additive (Figure 2). Molecular functions “protein binding” (GO:0005515) and “protein-containing complex binding” (GO:0044877) were also obtained.

In order to elucidate the location relative to the cellular structures in which the DEGs perform their function, several cellular components were identified in the functional network, representing the association between them (Figure 3). The analysis included the, “ribonucleoprotein complex” (GO:1990904; 10 up-regulated genes; 9 down-regulated genes), “vesicle” (GO:0031982; 20 up-regulated genes; 20 down-regulated genes), “transport vesicle membrane” (GO:0030658; 2 up-regulated genes; 5 down-regulated genes), “COPII-coated ER to Golgi transport vesicle” (GO:0030134; 2 up-regulated genes; 3 down-regulated genes), “Golgi-associated vesicle” (GO:000579810; 3 up-regulated genes; 4 down-regulated genes), “endosome” (GO:0005768; 10 up-regulated genes; 9 down-regulated genes), “vacuole” (GO:0005773; 9 up-regulated genes; 12 down-regulated genes), “lysosome” (GO:0005764; 7 up-regulated genes; 7 down-regulated genes), and “proton-transporting two-sector ATPase complex” (GO:0016469; 1 up-regulated genes; 3 down-regulated genes).

**Figure 4:**
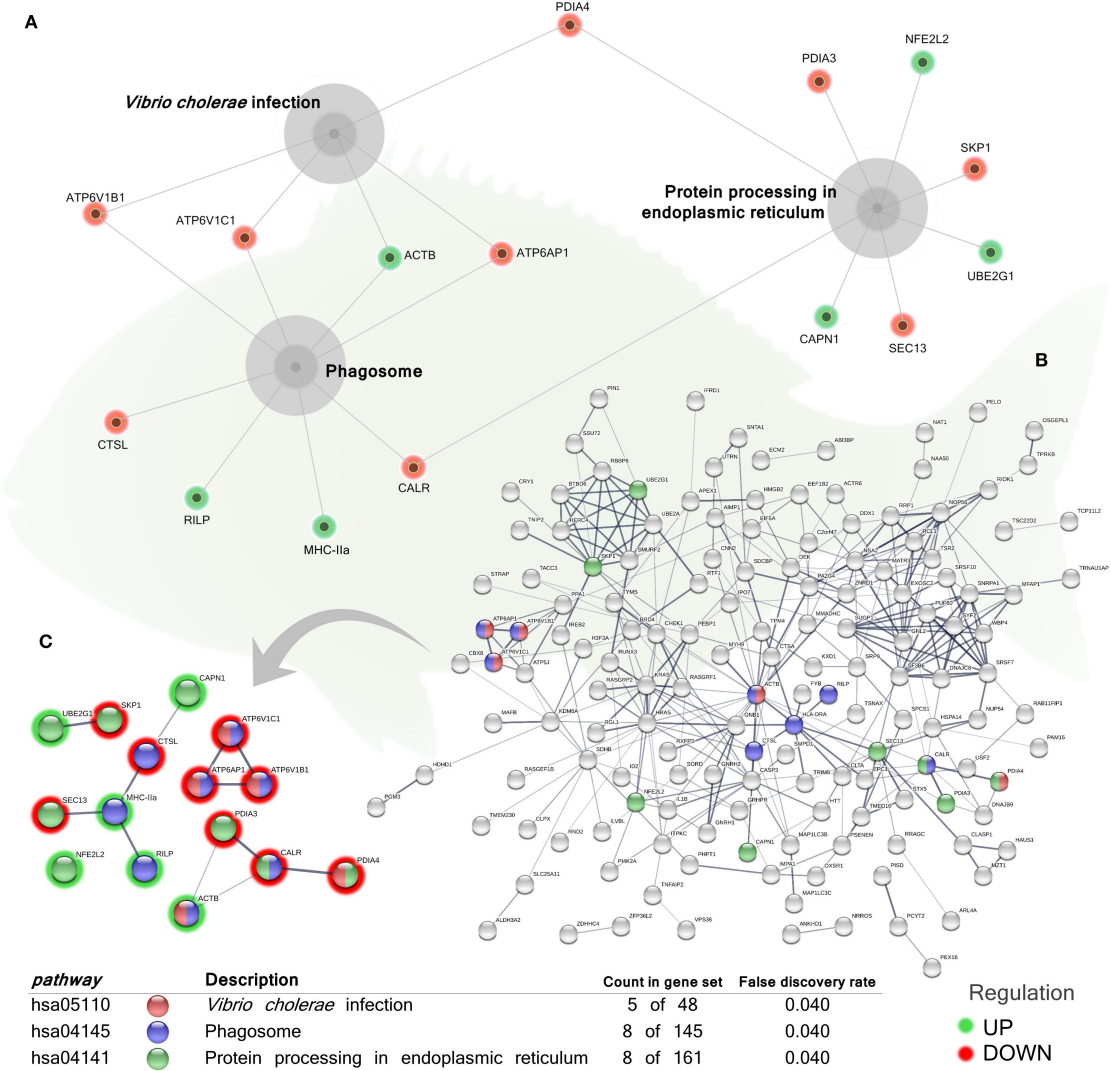
Kyoto Encyclopedia of Genes and Genomes (KEGG) pathways networks of the differentially expressed genes (DEGs) in the skin of juvenile gilthead seabream (*Sparus aurata*) fed a diet supplemented with a blend of garlic essential oil, carvacrol and thymol. **(A)** Gray cores represent the KEGG pathways significantly impacted by the DEGs obtained from the skin of fish fed the phytogenic-supplemented diet. Green-shaded nodes represent up-regulated genes and red-shaded nodes represent down-regulated genes. **(B)** Nodes colors indicate the KEGG pathways for each DEG represented within the overall transcripteractome. Pathways description, count of DEGs within each pathway and respective false discovery rate are described in the graphical figure legend. **(C)** Interactions exclusively among the DEGs within each KEGG pathway. Nodes colors indicate the KEGG pathways for each DEG represented. Pathways description, count of DEGs within each pathway and respective false discovery rate are described in the graphical figure legend. Green-shaded nodes represent up-regulated genes and red-shaded nodes represent down-regulated genes. For details, please refers to Supplementary Table 3.

In order to identify the pathways significantly impacted by the total DEGs obtained, the functional analysis of KEGG pathways revealed also significant differences in the regulation of genes associated with “protein processing in endoplasmic reticulum” (hsa04141; 3 up-regulated genes; 5 down-regulated genes), “phagosome” pathway (hsa04145; 3 up-regulated genes; 5 down-regulated genes), and “*Vibrio cholerae* infection” pathway (hsa05110, belonging to the “infectious disease: bacterial” group; 1 up-regulated gene; 4 down-regulated genes) in the skin of the group fed with the blend of tested phytogenics (Figure 4 and Supplementary Table 3).

**Figure 5:**
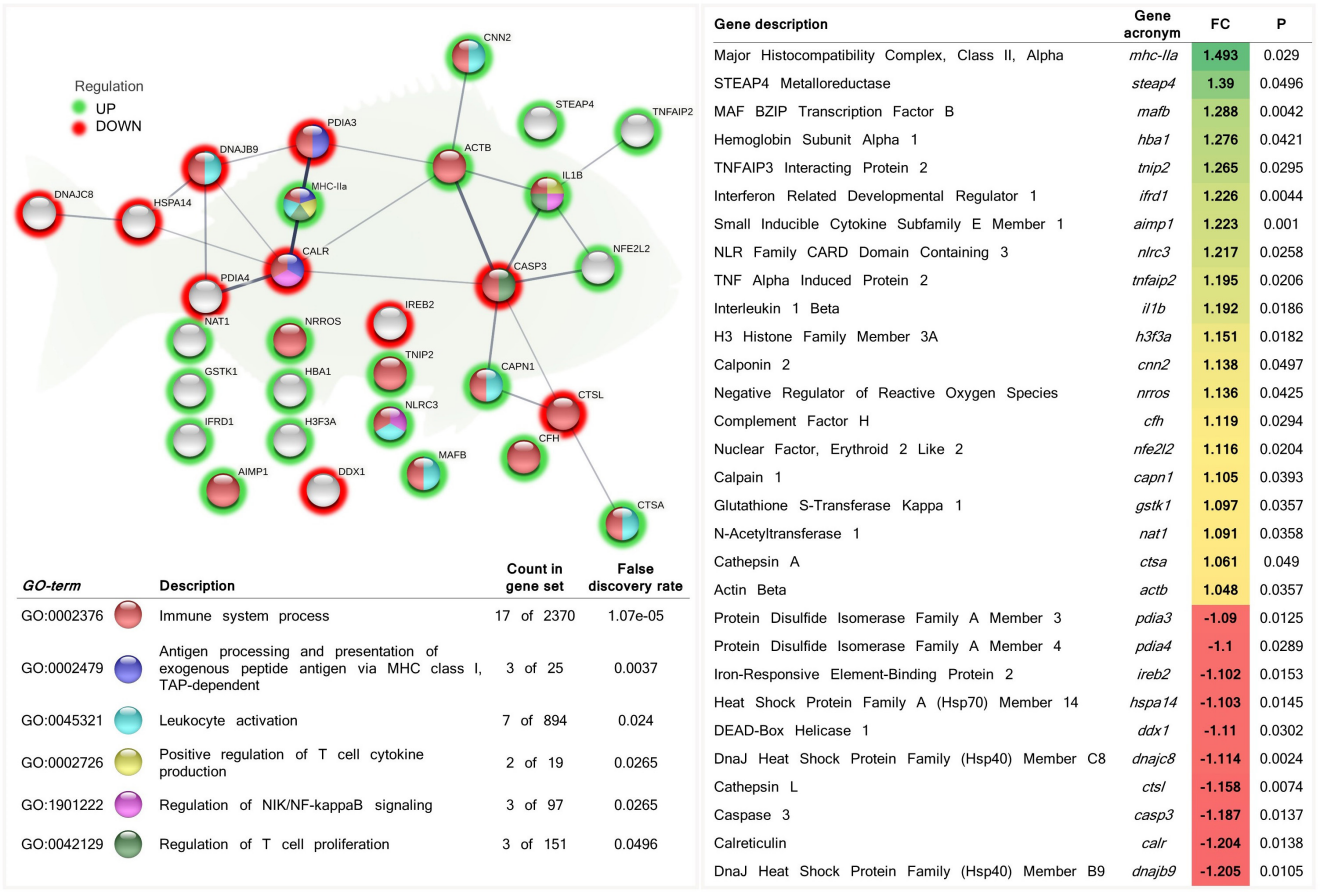
Functional biological network of differentially expressed genes (DEGs) recognized by their involvement in the immune response in the skin of juvenile gilthead seabream (*Sparus aurata*) fed a diet supplemented with a blend of garlic essential oil, carvacrol, and thymol. Green-shaded nodes represent up-regulated genes and red-shaded nodes represent down-regulated genes. Graphic keys and network stats: number of nodes = 30; number of edges = 24; average node degree = 1.6; average local clustering coefficient = 0.4; expected number of edges = 9; PPI enrichment *p*-value = 1.25e-05. Gene Ontology (GO) definition, count of DEGs within the biological process and respective false discovery rate are described in the graphical figure legend (bottom left). For details, please refers to Supplementary Table 4. Gene description, respective acronym, fold-change intensity (FC), modulation (color scale), and P-value are described (right).

Additionally, from the total DEGs obtained from the skin transcriptomic profile of fish fed the phytogenic-supplemented diet, a set of genes were selected by their involvement in the “immune system process” (GO:0002376; 12 up-regulated genes; 5 down-regulated genes). Among the processes related to immunity, “antigen processing and presentation of exogenous peptide antigen via MHC class I” (GO:0002479; 1 up-regulated genes; 2 down-regulated genes), “leukocyte activation” (GO:0045321; 6 up-regulated genes; 1 down-regulated genes), “regulation of NIK/NF-kappaB signaling” (GO:1901222; 2 up-regulated genes; 1 down-regulated genes), “positive regulation of T cell cytokine production” (GO:0002726; 2 up-regulated genes; 0 down-regulated genes), and “regulation of T cell proliferation” (GO:0042129; 2 up-regulated genes; 1 down-regulated genes) biological processes were highlighted (Figure 5 and Supplementary Table 4).

### Bacterial Growth Capacity on Skin Mucus

The transcriptome response arose the modulation of genes associated to immune processes involved in the response to infectious bacterial diseases. Thus, we evaluated whether such response implies a functional protective mechanism against pathogenic bacterial growth on skin mucus. Considering that our data registered a specific response to *Vibrio*, we included in our analysis the fish pathogen *V. anguillarum*. In addition, we also included as control *P. anguilliseptica* (another pathogenic marine fish bacteria) and *E. coli* as non-pathogenic fish bacterium.

**Figure 6:**
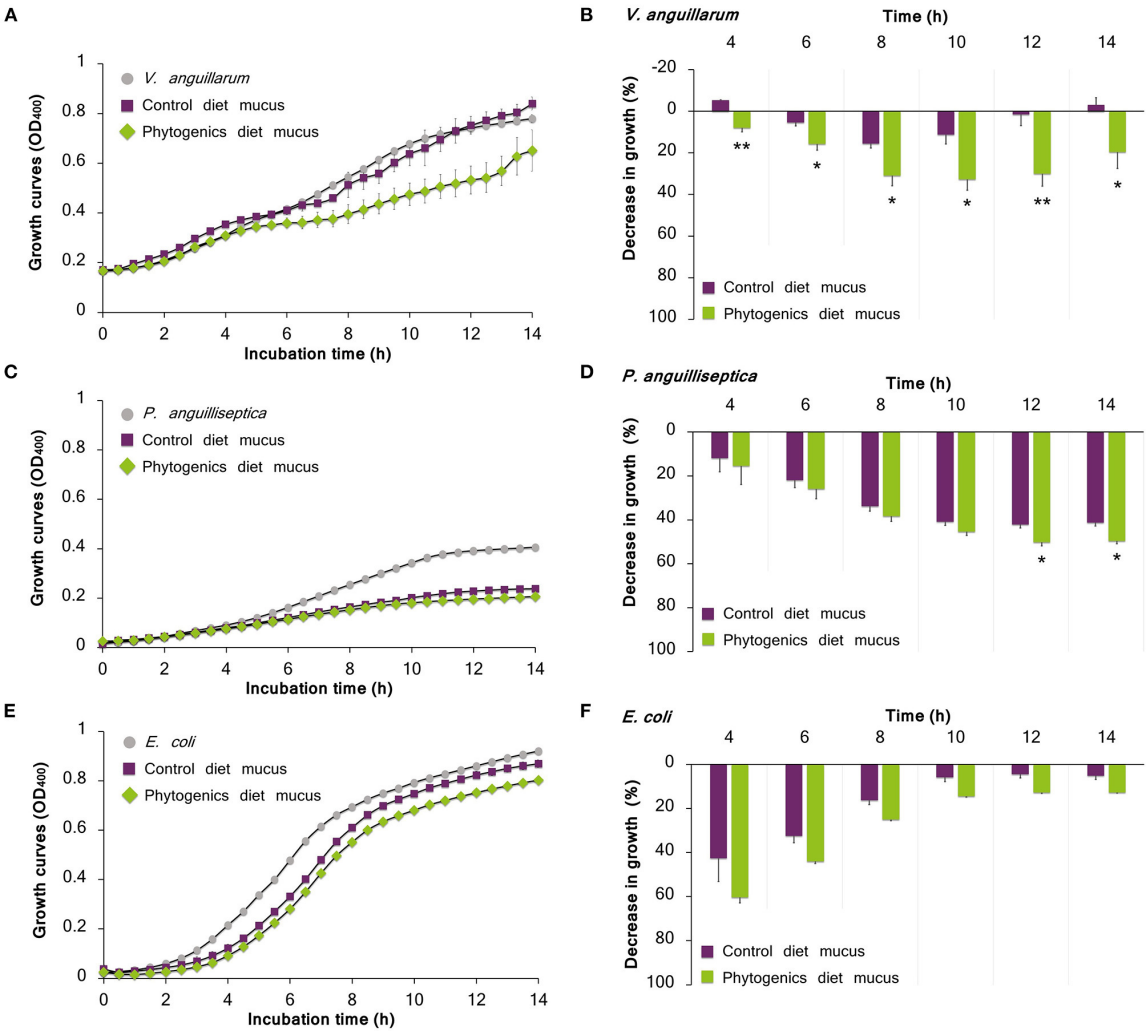
Bacterial growth on skin mucus of juvenile gilthead seabream (*Sparus aurata*) fed a diet supplemented with a blend of garlic essential oil, carvacrol and thymol, and a control diet devoid of the feed additive. Mucus samples were obtained from 3 pools of 6 individual fish. Data correspond to the mean ± SEM of triplicate bacterial growth curves of *V. anguillarum* **(A)**, *P. anguilliseptica* **(C)** and *E. coli* **(E)**. Gray circles correspond to bacteria growth in medium devoid of mucus; purple squares correspond to bacteria growth in the mucus of fish fed the control diet; and green rhombus correspond to bacteria growth in the mucus of fish fed the phytogenic-supplemented diet. Details on the statistical differences in bacterial growth between the experimental dietary groups is provided by the percentage of decrease in growth of *V. anguillarum* **(B)**, *P. anguilliseptica* **(D)**, and *E. coli* **(F)**. Asterisks indicate significant differences in bacterial growth between dietary groups (*t*-test, *P* < 0.05).

When cultured with the skin mucus from fish fed the phytogenic-supplemented diet, a reduction on the growth of the pathogenic bacteria *V. anguillarum* was observed (*t*-test; *P* < 0.05; Figures 6A,B). Growth decrease was recorded between 4 and 14 h of bacterial culture; the most accentuated decrease in growth values compared with control diet (over 30%) were found between 8 and 12 h (Figures 6A,B). Regarding *P. anguilliseptica*, a decline in bacterial growth was observed in both gilthead seabream skin mucus samples from fish fed the control and phytogenic-supplemented diets (Figures 6C,D). However, *P. anguilliseptica* growth decline was observed to be more accentuated in the mucus from fish fed the phytogenic-supplemented diet than in that of the control group at 12–14 h of culture (*t*-test; *P* < 0.05), with a maximum decrease in growth of 50.2 ± 1.6% at 14 h (Figure 6D).

Gilthead seabream mucus from both nutritional groups showed a decrease of *E. coli* growth during all the culture period (Figures 6E,F), though the most emphasized bacterial growth decrease was observed in the initial interval of 4–8 h with decreased growth value of 60.3 ± 2.5%, reducing gradually (Figure 6F).

### Mucus Stress Biomarkers

The skin mucus stress-related biomarkers and their ratios, as well as the ferric antioxidant power are summarized in Table 2. The content of soluble protein was not significantly affected by the functional feed additive (*t*-test, *P* > 0.05). However, glucose, lactate and cortisol levels were observed to be significantly lower in the mucus of gilthead seabream fed the phytogenic-supplemented diet (*t*-test, *P* < 0.05). No differences among dietary groups were observed in terms of the ferric skin mucus antioxidant power (*t*-test, *P* > 0.05). When data on skin mucus biomarkers were normalized with the protein content of the sample, only cortisol levels (cortisol/protein) were observed to be significantly reduced in skin mucus from fish fed the phytogenic-supplemented diet when compared to the control diet (*t*-test, *P* < 0.05). Metabolic aerobic response, measured in mucus as glucose/lactate ratio, was neither significantly affected by the dietary treatment.

**Table 2:** Skin mucus stress biomarkers of gilthead seabream fed an experimental diet supplemented with a blend of garlic essential oil, carvacrol and thymol, and the control diet devoid of the feed additive (*n* = 24 per dietary treatment).

	**Diet**	
	**Control**	**Phytogenics**
**Skin mucus stress biomarkers**		
Protein (mg/mL)	16.73 ± 2.22	12.86 ± 1.77
Glucose (μg/mL)	13.43 ± 3.95	6.88 ± 1.63*
Lactate (μg/mL)	9.80 ± 2.62	3.62 ± 1.09*
Cortisol (ng/mL)	3.47 ± 0.85	0.35 ± 0.05*
FRAP (μmol/mL)	1,923 ± 244	1,790 ± 315
**Skin mucus stress biomarkers ratios**		
Glucose/Protein (μg/mg)	0.74 ± 0.16	0.60 ± 0.17
Lactate/Protein (μg/mg)	0.46 ± 0.05	0.31 ± 0.12
Glucose/Lactate (mg/mg)	0.69 ± 0.14	0.77 ± 0.15
Cortisol/Protein (ng/g)	208 ± 48.80	31.85 ± 7.03**
FRAP/Protein (μmol/mg)	114 ± 16.9	139 ± 23.1

Asterisks indicate significant differences between experimental diets (*P < 0.05, **P < 0.01; t-test).

## Discussion

The fish skin mucosal surface is in direct contact with the aquatic environment and represents the first line of defense against external threats, determining pathogen adhesion to the epithelial surface (10, 12). As a mucosal tissue, the skin is characterized by its ability to produce mucus, which apart from being an intrinsic physical barrier, it contains glycosaminoglycans, lectins, antibacterial enzymes, immunoglobulins, and several structural, metabolic, stress-related, and signal transduction proteins [(23–25); among others]. In addition to mucus continuous secretion and replacement (26), its components provide an impermeable capacity against most bacteria and other pathogens, immobilizing them and inhibiting their proliferation before they can contact epithelial surfaces (10). Importantly, an intimate crosstalk between skin tissue and its exuded mucus in response to stimulus has been recently proposed (27), reinforcing the coordinated response capacity that takes place in this mucosal-associated lymphoid tissue.

In the present study, we evaluated the protective benefits of a blend of dietary garlic essential oil, carvacrol, and thymol on gilthead seabream in terms of skin transcriptome and skin mucus secretions. The potential increased skin mucus protective capacity observed in our gilthead seabream fed with phytogenic-supplemented diet could be attributed to the exudation of a variety of biologically active substances and several molecules of the innate and acquired immune system. In fact, many of them have an already reported biostatic and biocidal activities (28). Under this context, our transcriptomic analysis for the skin revealed that several genes coding proteins potentially involved in an enhanced skin mucosal protective capacity. For instance, the H3 Histone Family Member 3A gene (*h3f3a*) was up-regulated in the skin of fish fed the phytogenic-supplemented diet. Histones, full-length proteins and/or fragments, are recognized antimicrobial molecules (29), which are found in the mucus of several fish species (30, 31), including gilthead seabream (32, 33). Calpain 1 (*capn1*), a non-lysosomal cysteine protease, was observed to be up-regulated by the functional additive as well. Several calpain proteins were mapped in gilthead seabream's epidermal mucus (34) while its presence in the skin mucus of cod (*Gadus morhua*) was suggested to be a key protective element against *V. anguillarum* infection (35).

Iron is an essential micronutrient required for most bacteria to grow; in the host, this metal is associated to iron-binding proteins, such as transferrin found in fish skin and mucus (23, 30, 32), limiting its availability to invading pathogenic bacteria (36). For instance, *V. anguillarum* iron-uptake system is crucial for sequestering iron from these proteins and accomplishing skin colonization and penetration (37). Interestingly, some genes related with iron metabolism were observed to be down-regulated in the skin of fish fed the functional diet. In our transcriptional analysis, the Iron-Responsive Element-Binding Protein 2 (*ireb2*) gene was down-regulated in fish fed the phytogenic-supplemented diet. When cell's iron levels are low or depleted, this RNA-binding protein binds to iron-responsive elements, found for instance in transferrin mRNAs, regulating the translation and stability of those mRNAs and consequently regulating iron availability (38). In zebrafish, the increase of *ireb2* expression in spleen was proposed to be linked to an augmented iron uptake from *V. anguillarum* (39). Hence, the down-regulation of *ireb2* observed in the skin of fish fed the phytogenic-diet could be suggesting a decrease in the cellular iron uptake as a consequence of the reduction of pathogenic bacteria load in the mucosal tissue. This hypothesis is supported by the decreased growth capacity for pathogenic bacteria in skin mucus observed in the fish fed the tested functional diet.

On the other hand, iron plays an important role in hemoglobin production. The Hemoglobin Subunit Alpha (*hba1*) was up-regulated in the skin of fish fed the phytogenic-supplemented diet. Hemoglobin functionality is not restricted to oxygen transport, since it also binds to pathogen-associated molecular patterns (PAMPs), triggering an immune Toll-like receptor (TLR)-mediated signal transduction (40). In addition, the STEAP4 Metalloreductase (*steap4*) transcripts increased by the phytogenic-diet. This metalloreductase is involved in iron and copper homeostasis, playing also a role in the protection against inflammatory-mediated cellular damage (41). In Atlantic salmon (*Salmo salar*) skin, *steap4* was up-regulated by dietary phytogenics, which was associated to iron sequestration, inflammation and an increased protective capacity against lice infection (42). The *hba1* and *steap4* up-regulation in the skin observed in our study may be implicated in a reduction in iron availability in the skin surface and mucus of fish fed the phytogenics, hampering pathogenic bacteria growth as observed in the potential mucus antibacterial capacity observed in our study.

Fish SALT is characterized by both humoral and cellular components which intimately communicate to mount an immune response in which both innate and adaptive defense mechanisms are involved (15). For instance, the Interferon Related Developmental Regulator 1 (*ifrd1*) was up-regulated by the functional diet. *Ifrd1* gene encodes a protein related to interferon family. In fish is widely recognized by its involvement in the innate immune antiviral response (43), while it is also highly expressed in differentiating neutrophils, playing an important role in neutrophils effector function (44). An increase of *ifrd1* transcripts was observed in the skin of zebrafish (*Danio rerio*) infected by *Aeromonas hydrophila* (45), whereas its up-regulation was also related to its role in the immediate response of the fish immune system to stress (46). Moreover, transcripts of the Complement Factor H (*cfh*) increased in the skin of gilthead seabream fed the phytogenic-supplemented diet. CFH, a major regulator of the complement system, is essential for directing the complement system toward pathogen-related infections, since its transcription is induced by lipopolysaccharide (LPS) (47), whereas it increases the contact between neutrophils and pathogens, increasing cell's phagocytosis capacity and antimicrobial activity (48). CFH is also reputed for protecting host cells and tissues from the self-innate immunity (49). This occurs due to the interaction of the factor H with the C3 convertase and the C3b component (50). Although CFH is predominantly expressed in the liver compared to other tissues and organs like the muscle, intestine, fins, eyes, and gills (47), present data indicate that the skin may also play a relevant role in the regulation of the alternative pathway of complement in skin secretions. This data is in agreement with different studies that have identified several complement factors in fish skin mucus secretions for several species (30, 51), including gilthead seabream (23, 24).

Our study also revealed that Cathepsin A (*ctsa*) was up-regulated in the skin of fish fed the phytogenic-supplemented diet. An increase in turbot (*Scophthalmus maximus*) *ctsa* expression in skin was described in response to infection challenges, while *ctsa* genes microbial binding capacity was also observed, in which a high affinity to LPS, and a lower affinity to lipoteichoic acid (LTA) and peptidoglycan (PGN), was suggested to be implicated in the sensing and phagocytosis of bacterial pathogens (52). By contrast, in our study the expression of Cathepsin L (*ctsl*) was down-regulated. Different cathepsins have been detected in the mucus of several fish species, which were observed to exhibit high bacteriolytic activity against several fish pathogens (53, 54), evidencing their key role in fish mucosal innate immunity. Fish skin *ctsl* transcripts were observed to be significantly up-regulated after challenges with several bacterial pathogens, including *V. anguillarum* (54, 55). Moreover, as for *ctsa* genes, it was demonstrated that *ctsl* genes have strong *in vitro* binding capacity to microbial ligands, suggesting an important role of *ctsl* in fish mucosal immunity (55). The different gene expression pattern observed for *ctsa* and *ctsl* could be related to a time-dependent response, as suggested previously for genes involved in the immune response in fish subjected to feeding trials (56). The differential expression between both cathepsins could be also attributed to the mucosal tissue response specificity. In this way, it has been reported that the same stimulus may differentially modulate the expression for the same genes depending of the mucosal tissue evaluated (57). However, if both genes are linked with the decrease in the bacterial growth observed at skin mucus deserves further investigations.

Fish professional phagocytes include macrophages, granulocytes, dendritic cells and B cells, and as for other vertebrates, phagocytosis in fish is recognized as a critical component of the innate and adaptive immune responses against pathogens, known to elicit several antimicrobial mechanisms. Under this context, the KEGG “Phagosome” pathway obtained from our functional analysis suggests the modulation of phagocytic events by the administered phytogenics. For instance, despite the extracellular roles of cathepsins (58), these proteins are mainly found in endolysosomal structures where they are crucial for protein degradation and Major Histocompatibility Complex (MHC) Class II mediated immune responses (59). Interestingly, the MHC Class II Alpha gene (*mhc-IIa*) was the second most up-regulated gene in the skin of fish fed the phytogenic-supplemented diet. While MHC-IIa protein was identified in gilthead seabream skin mucus proteome (34), the main function of fish MHC Class II molecules is to present the peptides generated in the endolysosomal structure on the cell surface of B cells and phagocytes for their recognition by the CD4+ T cells (60). In fact, the gilthead seabream acidophilic granulocytes, considered the main professional phagocytic cell type for this fish species, were demonstrated to show high *mhc-IIa* gene expression (61). Moreover, they have also proved to have phagocytic activity against bacterial pathogens such *V. anguillarum* (62), being able to release antimicrobial peptides into the phagosome of the ingested pathogenic bacteria (63). The “leukocyte activation”, “regulation of T cell proliferation”, and “antigen processing and presentation of exogenous peptide antigen via MHC class I” biological processes obtained from our enrichment analysis, might suggest the activity of acidophilic granulocytes and/or other immune cells in the skin of gilthead seabream fed the functional diet. Similarly, previous transcriptional results on the effect of the same functional feed additive in gilthead seabream mucosal tissues such gills (17) suggested the recruitment and activation of acidophilic granulocytes as a consequence of the immunostimulatory effect of this additive.

The functionality and modulation of genes related to the “endosome” and “lysosome” cellular components reinforce the hypothesis of an increased professional phagocytic activity in the skin of fish fed the phytogenic-supplemented diet. Among them, Rab-interacting proteins coding genes (*rilp, rab11fip1*) were up-regulated. These genes are involved in several processes like (i) endosomal recycling (64), (ii) endocytic transport to degradative compartments (65) and (iii) in the control of membrane trafficking along the phagocytic pathway (66). In addition, the Microtubule Associated Protein 1 Light Chain 3 Gamma (*map1lc3c*) was up-regulated. This autophagy-related protein is involved in the LC3-associated phagocytosis, in which LC3 is recruited to the phagosome membrane during phagocytosis of pathogens, enhancing the fusion between phagosome and lysosomes (67). In fact, fish epidermal macrophages are characterized by well-developed endoplasmic reticulum and Golgi areas and several lysosome-like vesicles and phagosomes (68). Remarkably, the up-regulation of the MAF BZIP Transcription Factor B (*mafb*), a myeloid lineage-specific transcription factor, which expression levels increase during macrophage differentiation and maturation (69), was also observed in the skin of fish fed the phytogenic-supplemented diet. Therefore, the regulation of LC3 proteins-coding genes and the up-regulation of *mafb* by the phytogenics might support the participation of phagocytic cells in the immune response from the skin observed in our study. The down-regulation of Microtubule Associated Protein 1 Light Chain 3 Beta (*map1lc3b*) opens the possibility to selective and differential mechanisms of activation aimed to the promotion of the phagocytic activity in response to phytogenic supplementation.

Garlic, carvacrol, and/or thymol have been several times described to improve immune cells phagocytic capacity. For instance, dietary garlic (0.5 and 1% inclusion) enhanced the activity of head kidney macrophages phagocytic in rainbow trout (*Oncorhynchus mykiss*) (70). Similar results were observed for blood leukocytes of juvenile hybrid tilapia (*Oreochromis niloticus* x *Oreochromis aureus*) fed a 0.5% garlic-supplemented diet (71). Likewise, carvacrol and thymol supplementation (0.2%) in juvenile hybrid tilapia's diet significantly enhanced phagocytosis of head kidney macrophages (72). The phagocytic activity of serum leukocytes from common carp (*Cyprinus carpio*) was increased due to dietary oregano's essential oil, which is rich in carvacrol and thymol, in a dose-dependent manner (73). A similar enhanced head kidney leukocytes' phagocytosis was also found in gilthead seabream fed a diet supplemented with oregano powder (0.5 and 1%) (74).

Phagocytic events are driven by rearrangements of the actin cytoskeleton (75). Calponin 2 (*cnn2*), an actin cytoskeleton-associated regulatory protein that restricts the pro-inflammatory activation of macrophages (76), was up-regulated in the skin by the phytogenics. Similarly, the Actin Beta (*actb*) gene was also up-regulated. Actin is commonly found in the mucus of several fish species, including gilthead seabream (23, 32), which has led to speculations on its immune function in fish defense. In fact, ACTB levels were observed to be significantly increased in sea lice challenged Atlantic salmon (30, 51). In gilthead seabream, *actb* expression in skin mucus was favored by a dietary probiotic administration (24). Furthermore, the extracellular cytoplasmic actin in insects was observed to bind to bacteria surface, mediating its phagocytosis and killing (77), suggesting that actin could be functionally active in fish skin mucus as well. However, this hypothesis needs further investigation. In summary, our transcriptional analysis could be indicating an enhanced phagocyte function in the skin of fish fed the phytogenic-diet, which would suggest the promotion of the host's defense ability in resisting bacterial infections.

In order to prevent the stable colonization of potential pathogens, mucus is continuously synthesized, secreted and replaced (26). This continuous regulation of mucus secretions represents one of the first barriers against potential pathogens and toxins (10). Accordingly, our functional analysis determined the modulation of the KEGG “Protein processing in endoplasmic reticulum” pathway and several cellular components connected to the secretory pathway, such as transport vesicles, Golgi-associated vesicles and vacuoles. The regulation of the secretory machinery could be supporting the active biosynthesis and release of immune-related factors on the skin mucus that would mediate in the response observed in our study. The mucus secretion is a complex process that represents the endpoint of the interaction between the innate immune system, endocytosis and autophagy events, ROS generation and mucin secretion (78). From the immunological point of view, cytokines, and chemokines are molecules trafficked in secretory granules and vesicles through secretory pathways in immune cells (79) that could be also involved in the response observed. Under this context, transcripts of the pro-inflammatory cytokine Interleukin 1 Beta (*il-1*β) were observed to be increased in the skin of fish fed the phytogenic-supplemented diet. In fish, IL-1β is a recognized chemoattractant for leukocytes (80). Additionally, the Aminoacyl tRNA Synthetase Complex Interacting Multifunctional Protein 1 (*aimp1*) gene was also up-regulated. Secreted AIMP1 possesses inflammatory cytokine activity responsible for activating monocytes and inducing the production of pro-inflammatory cytokines, mainly the Tumor Necrosis Factor (TNF) (81). In accordance, genes that are activated in response to the pro-inflammatory cytokine TNF, such the TNF Alpha Induced Protein 2 (*tnfaip2*) and TNFAIP3-Interacting Protein 2 (*tnip2*) were also up-regulated by the phytogenics in our study. The last inhibits the NF-kB pathway activation (82), negatively regulating the transcription of other pro-inflammatory cytokines and, consequently, controlling the inflammatory response. Thus, such response could be intimately related with the role of AIMP1 in dermal fibroblast proliferation and wound repair (83).

Other mediators of the inflammatory response were also observed to be regulated in the skin of fish fed the phytogenic-diet. For instance, the Negative Regulator of Reactive Oxygen Species (*nrros*) gene expression was increased. The NRROS protein regulates ROS production by phagocytes during inflammatory response, allowing phagocytes to produce high amounts of ROS in case of infection, while minimizing host's tissue damage (84). NRROS is also suggested to play a role in the maintenance of the immune homeostasis through the inhibition of TLR-mediated MAPK and NF-kB activation (85). Another negative mediator of the inflammatory response, the NLR Family CARD Domain Containing 3 (*nlrc3*) was also observed to be up-regulated in fish fed the phytogenic-supplemented diet. NLRC3 is known to negatively regulate NLR-mediated inflammatory responses (86). In several fish species, the overexpression of *nlrc3* was observed to be systematically induced by bacterial and LPS challenges (87, 88), including in mucosal tissues (89), demonstrating its important role in the fish innate immune response and homeostasis maintenance. The DEAD-Box Helicase 1 (*ddx1*), reported to enhance NF-kB mediated transcriptional activation (90) and recently associated to antiviral responses in fish (91), was also down-regulated in the skin of fish fed the phytogenic-supplemented diet. Therefore, according to the overall response observed considering the transcriptomic profiling of *il-1*β, *aimp1, tnfaip2, tnip2, nrros, nlrc3*, and *ddx1*, the regulation of these pro- and anti-inflammatory genes suggests an active cytokine secretion, an immune cell-cell signaling, and the tight control of such response.

Regardless of its critical function protecting the host, the skin mucus also represents an important portal of entry for pathogens since it can provide a favorable microenvironment for some bacteria, the main disease agents for fish, which may induce the development of biofilms depending on the pathogen adhesion capacity (10). Interestingly, the down-regulation of genes of the KEGG “*Vibrio cholerae* infection” pathway was obtained from the analysis of the DEGs of fish fed the phytogenic-based additive. Although the analysis was performed using *Homo sapiens* as model organism, the down-regulation of several genes of the “*Vibrio cholerae* infection” KEGG pathway could also be applied, from a comparative point of view, to an infection process involving a *Vibrio* species in a mucosal tissue, such as *V. anguillarum*, as a consequence of the protective effect of the additive. For instance, the down-regulation of several coding genes for V-type H+ ATPase subunits (*atp6v1c1, atp6v1b1, atp6ap1*) in fish fed the phytogenic-supplemented diet was obtained. The V-type H+ ATPase complex in endosomes and lysosomes is responsible for the vesicle import of protons and maintenance of the internal acidic pH, crucial for degradative enzymes activity (92). Apart from the “proton-transporting two-sector ATPase complex” cellular component obtained from our enrichment analysis, the down-regulation of the above-mentioned genes also figured in the KEGG “Phagosome” pathway, which could be suggesting a decreased phagosomal activity at the sampling time evaluated (65 days of diet administration). Such gene modulation could be associated to a decrease in skin bacterial pathogens in our fish fed with phytogenic-supplemented diet. This hypothesis is in agreement with the results obtained from the bacterial growth assessment in the epidermal mucus *in vitro*.

Bacterial growth in gilthead seabream mucus was evaluated for both dietary groups, control and phytogenic-supplemented diets, using two pathogenic fish bacteria, *V. anguillarum* and *P. anguilliseptica*, as well as a non-pathogenic fish bacterium, *E. coli*. Our study revealed that the tested feed additive reduced bacteria growth capacity, suggesting an enhanced skin mucus inhibitory capacity against both *V. anguillarum* and *P. anguilliseptica*. The chosen pathogenic bacteria are widely recognized as disease causing agents in several fish species (93, 94), including gilthead seabream (95). The observed reduced growth capacity of *V. anguillarum* in the skin mucus of gilthead seabream fed the phytogenic-supplemented diet is especially relevant because of the evidence that *Vibrio* strains exhibit a chemotactic response to mucus (37). In the last years, disease records indicate that *Vibrio* spp. infections are the most common bacterial infections in gilthead seabream, mostly reported during the hatchery phase (96). Besides, *P. anguilliseptica* is one of the main agents responsible for outbreaks associated with “winter disease” in gilthead seabream farming, being considered a more opportunistic pathogen whose infections in gilthead seabream usually occur when fish are under environmental stress (97). The different growth curves behavior of *V. anguillarum* and *P. anguilliseptica* in gilthead seabream mucus may be attributed to different virulence of pathogenic bacteria, differences in chemotaxis to skin mucus and adherence capacity.

Our results suggest that the phytogenic-supplemented diet may reduce the settlement of the studied bacteria in the skin surface, decreasing the risk of infection. Previous nutritional studies in which diets containing garlic, carvacrol, or thymol provided effective mucus antibacterial characteristics against fish pathogens. For instance, dietary garlic supplementation (5 and 10 g kg-1) was demonstrated to significantly increase skin mucus antimicrobial activity against several bacterial pathogens in the freshwater Caspian roach (*Rutilus rutilus*) (98). A similar increased bactericidal activity against *Photodamselae* subsp. *piscicida* was observed in the skin mucus of gilthead seabream juveniles fed diets supplemented with oregano powder (0.5 and 1% inclusion) for 15 and 30 days (74). In addition, an enhanced skin mucus bactericidal activity against *V. parahaemolyticus* and *Aeromonas hydrophila* was observed in Pacific red snapper (*Lutjanus peru*) fed a diet supplemented with a medicinal plant extract rich in carvacrol and thymol (0.5, 1, and 2% inclusion) (99). On the contrary, other studies incorporating *Oliveria decumbens*, which is rich in carvacrol (18.8 and 52.9% included in an essential oil and aromatic water fractions, respectively) and thymol (20.3 and 37.6% included in an essential oil and an aromatic water fractions, respectively), in Nile tilapia (*O. niloticus*) diets reported the absence of changes in mucus bactericidal activity against *Streptococcus iniae*. By contrast, the compounds showed high antibacterial capacity when evaluated *in vitro* (100). Similarly, Beltrán et al. (74) did not find an enhancement of antibacterial activity against *V. anguillarum* in the mucus of gilthead seabream fed a diet supplemented with *Origanum vulgare* at 0.5 and 1.0%, although a significant increased bactericidal activity against *Photobacterium damselae* subsp. *piscicida* was observed. The above-mentioned contradictory results could be a consequence of different factors, such as different phytogenics and their content in bioactive compounds, supplementation period of the functional diet, dietary compounds delivery and bioavailability, or an evidence of the synergy between the phytogenics tested that might improve such bactericidal capacity.

Moreover, the *E. coli*, a non-pathogenic bacterium for fish, was also used as an indicator of the potential antibacterial capacity of the skin mucus, neglecting a potentially acquired immunization. Although no significant differences were observed among the experimental diets, a decreasing trend in *E. coli* growth was observed due to the presence of mucus from both dietary groups in the culture medium. Interestingly, garlic-supplemented diets caused a significant increase in the Caspian roach fry skin mucus antibacterial activity against *E. coli* when compared to the control group (98). In addition, carvacrol and thymol were reported to have a bacteriostatic and bacteriolytic *in vitro* activity against most Gram positive and negative bacteria, including *E. coli* (101). Nevertheless, the absence of an inhibitory response against *E. coli* growth in our study could be suggesting the promotion of the skin innate immunity against fish pathogens (102).

From a physiological perspective, bacterial pathogens such *V. anguillarum* are able to elicit strong cortisol-mediated stress responses when they adhere to the mucosal surface (36). For this reason, in this current study we also measured classic skin mucus stress biomarkers in order to establish a correlation between the gene expression profile, antibacterial response and the fish physiological status. Therefore, only cortisol was observed to be significantly reduced in fish fed the phytogenic-supplemented diet. Although the exact mechanisms involved in cortisol exudation through fish mucus are still unclear, cortisol is the main glucocorticoid and the final product of the HPI axis response to stress, varying considerably among species and according to the duration and severity of the stressor (103). Cortisol decrease also favors the fish local mucosal immunity, promoting more effective defense responses against pathogens (104). Besides the hypothesized decrease in skin pathogenic bacteria and its potential impact on cortisol-induced responses, and *vice versa*, several phytogenic active substances have been reported to have sedative properties in fish (105). For instance, garlic powder inclusion in common carp diet (0.5, 1, and 1.5%) decreased plasma cortisol and glucose levels, mitigating ammonia stress-induced effects (106). Similar results were obtained in rainbow trout fed 3% garlic powder supplemented diets (107). Accordingly, the dietary supplementation of a similar additive containing garlic and Labiatae plant essential oils (0.02% inclusion) reduced significantly plasma cortisol levels in European seabass (*Dicentrarchus labrax*) challenged by confinement (108). Although the diet effect on fish stress response is usually evaluated in blood, a positive correlation between cortisol levels on plasma and fish mucus was demonstrated (109, 110) including for gilthead seabream (111). Therefore, the observed decrease in mucus cortisol may suggest a decrease in the allostatic load due to the properties of the phytogenics used in this current study and/or as a consequence of the promotion of the non-specific innate immunity, although these two hypotheses are not mutually excluding.

During stress adaptation, cortisol has been suggested as a signal factor that induces tissue specific molecular programming in fish (112). Cortisol is able to induce a skin local stress response in fish (57, 94, 109, 113), which is particularly characterized by the increase of the secretory activity, related vesicles, apoptosis (114), and transcriptional alterations (57). Under this context, the observed changes in mucus cortisol secretion might be contributing to the obtained secretory-related transcriptional response as well. Furthermore, since a correlation between cortisol secretion and skin mucus oxidative stress was demonstrated (115), a reduction of the skin oxidative stress in response of an increase in antioxidative power induced by the tested phytogenics would be also expected. In fact, in our current study genes coding for antioxidative enzymes were observed to be up-regulated in the skin of fish fed the phytogenic-supplemented diet, as for example the Glutathione S-Transferase Kappa 1 (*gstk1*). GSTK1 belongs to the as Glutathione S-Transferase (GST) superfamily of oxidative stress enzymes, which are mainly known for their important role in cellular detoxification (116). GSTs have been used as markers for fish antioxidative capacity (117), including the evaluation of phytogenics in aquafeeds (118). Under stress-imposed conditions or injury, the transcription of skin *gst* is usually decreased and associated to immunosuppression in gilthead seabream (33). The Nuclear Factor Erythroid 2 Like 2 (*nfe2l2*), an important transcription factor that positively regulates the expression of cytoprotective genes (119), and the N-Acetyltransferase 1 (*nat1*), known for its participation in the detoxification of drugs and other xenobiotics (120), were also observed to be up-regulated by the functional diet. In addition, Heat Shock Protein family genes (*dnajc8, dnajb9*, and *hspa14*) were down-regulated in the fish fed the phytogenic-supplemented diet. In particular, HSPA14 is member of the Hsp70 family, which proteins levels have been described to increase in fish under stress or pathological conditions (121, 122). Therefore, the regulation of these genes is supporting the involvement of immune cells in the skin response observed, suggesting a reduction of the skin oxidative stress in response of the reduced mucus cortisol secretion and/or by the antioxidative characteristics of the tested phytogenics. In fact, the inclusion of garlic (106, 123), carvacrol, and thymol (73, 124) in aquafeeds have been continuously demonstrated to enhanced fish antioxidant status. In the present study, the epidermal mucus antioxidant capacity was also measured by mean of the FRAP analysis (102, 115). Although our transcriptional analysis revealed the regulation of several markers of oxidative stress, according to FRAP's analyses the mucus antioxidant power was not significantly changed by the tested additive. Our results are in agreement with some previous studies which reported that the dietary supplementation of carvacrol and/or thymol-rich compounds did not affect the skin mucus biochemical contents or mucus antioxidant status (74, 100).

Collectively, these data clearly suggest a relationship between the tested phytogenics, the increased skin innate immunity and a cortisol-mediated response, promoting the overall animal welfare. In fact, according to the only biological process significantly regulated among the experimental diets, the RNA processing biological process, several genes implicated in ribosomal proteins synthesis (*riok1, rcl1, rrp1, nop56, nsa2, tsr2*) were mainly up-regulated in fish fed the phytogenic-supplemented diet. Since ribosome biogenesis is the cell's most costly process in terms of energy expenditure, this process must be tightly regulated in order to avoid wasted energy (125). Consequently, the up-regulation of such genes could be suggesting less stressed cells able to direct their energy into this process. Since skin from the control group appear to be more susceptible to be colonized by pathogenic bacterial strains, it could be spending more energy in defense mechanisms and bacterial clearance than fish fed the phytogenic-supplemented diet.

In summary, our analysis of the skin transcriptional profiling as well as the skin mucus biomarkers and lower pathogenic bacterial growth capacity revealed a multifactorial response to the dietary administration of garlic essential oil, carvacrol, and thymol, mainly through the transcriptional regulation of factors of the innate immunity and the stimulation of the secretory pathway. Our results suggest that the phytogenic-supplemented diet induces the activation of the mucosal immune response that promotes the secretion of non-specific immune molecules into the skin mucus, resulting in the decrease of bacterial growth capacity in mucus. From our transcriptomic enrichment analysis, the regulation of genes related with the secretory pathway suggests that the tested phytogenics could be also stimulating the recruitment of phagocytic cells. The reduction in skin mucus cortisol is in line with the recognized properties of the phytogenics. Since the exact mechanisms promoted by the tested phytogenics were not yet demystified, further analysis should be made in order to assess the effect of the experimental diet on skin phagocytes and phagocytosis potential. More efforts are also needed for determining the impact of the functional additive on the skin defensive status against other pathogenic bacteria that threaten the success of aquaculture under intensive farming regime.

## Data Availability Statement

The datasets presented in this study can be found in online repositories. The names of the repository/repositories and accession number(s) can be found in the article/Supplementary Material.

## Ethics Statement

IRTA facilities, where the trial was conducted, are certified and have the necessary authorization for the breeding and husbandry of animals for scientific purposes. All procedures involving the handling and treatment of the fish were approved as far as the care and use of experimental animals are concerned, by the European Union (86/609/EU), the Spanish Government (RD 1201/2005), and the University of Barcelona (Spain).

## Author Contributions

Biological samplings were performed by EG, JF, RS, LF-A, IS, and AI. The transcriptomic data analysis and interpretation was performed by JF, RS, EV-V, and FER-L. LF-A, IS, and AI carried out the skin mucus analyses. The study was supervised by EG, FER-L, and LT. JF wrote the original draft. Funding was obtained by EG and LT. All the authors provided critical feedback, read, and agreed to the published version of the manuscript.

## Conflict of Interest

JF is a current TECNOVIT-FARMFAES S.L employee conducting an Industrial Ph.D. The remaining authors declare that the research was conducted in the absence of any commercial or financial relationships that could be construed as a potential conflict of interest.
